# Genital herpes and genital warts affect quality of life and emotional well-being

**DOI:** 10.1177/09564624261438901

**Published:** 2026-03-28

**Authors:** Maja af Klinteberg, Alma Seyer, Nirina Andersson

**Affiliations:** 1 Department of Public Health and Clinical Medicine, Dermatology and Venereology, 174459 Umeå University, Umeå, Sweden

**Keywords:** genital herpes, genital warts, health-related quality of life, RAND-36, psychological distress, sexually transmitted infections, Sweden

## Abstract

**Background:**

Health-related quality of life is an important outcome in sexually transmitted infections, but data on genital herpes and genital warts in Sweden are limited. This study aimed to describe health-related quality of life in these patient groups at a Swedish STI clinic and to compare their scores with population norms and between the two diagnoses.

**Methods:**

We conducted a clinic-based cross-sectional survey among adults with clinician-confirmed genital herpes or genital warts at an STI clinic in northern Sweden between November 2023 and September 2025. Health-related quality of life was measured using RAND-36. Global quality of life and sexual life were rated on visual analogue scales, and psychological symptoms were assessed with the DASS-21.

**Results:**

In total, 91 patients were included, 45 with genital herpes and 46 with genital warts. Current visible lesions were more common in genital warts (98%) than in genital herpes (45%). No significant differences were observed between the groups in global quality of life, sexual life ratings and DASS-21 (all *p* > 0.12). In contrast, genital herpes patients scored lower than genital warts patients in RAND-36 domains: vitality (46.1 vs 56.7, *p* = 0.034); and social functioning (68 vs 79, *p* = 0.048). Compared with Swedish norms, genital herpes patients scored 15-27 points lower in mental and social domains (all *p* < 0.001), and 9 points lower in general health (*p* = 0.015). Genital warts patients scored 18 points lower in role emotional (*p* = 0.008).

**Conclusions:**

Genital herpes and genital warts were both associated with impaired health and quality of life in this Swedish clinical sample. Genital herpes patients demonstrated greater mental and social impairment compared to those with genital warts and norms. These findings highlight the need for psychosocial assessment, clear information on transmission and recurrences, and targeted support for patients with pronounced psychological distress.

## Introduction

Health-related quality of life (HRQoL) is an important outcome in sexually transmitted infections (STIs), as symptoms, recurrence patterns and social stigma may substantially affect daily functioning, intimate relationships and psychological well-being. Genital herpes and genital warts are common STIs that illustrate this complexity. While many individuals remain asymptomatic or experience mild disease, others suffer from recurrent or persistent symptoms, with a highly variable impact on quality of life (QoL).^1,2^

Importantly, the perceived burden of genital herpes and genital warts does not necessarily correspond to the severity or frequency of physical symptoms. Previous studies have shown that individuals with few or no visible lesions may still report considerable psychological distress, including anxiety, shame and concerns related to sexual relationships.^1,3^ Concerns regarding transmission risk and potential consequences for partners may lead to avoidance behaviours, impaired sexual life and ongoing psychological distress.^1,2^

From a clinical perspective, genital herpes and genital warts differ in their natural history and management. Genital herpes is characterised by lifelong latency with recurrent episodes that often decrease in severity over time. Antiviral therapy can be used episodically or as suppressive treatment and has been shown to reduce transmission risk.^4^ Genital warts lesions may resolve spontaneously, but recurrences are common, and treatment often requires repeated local or destructive interventions.^2^

Despite the high clinical relevance of genital herpes and genital warts, data on HRQoL in affected individuals are limited, particularly in a Swedish context. National surveillance focuses on incidence rather than patient-reported outcomes, and findings from other countries may not be directly applicable due to differences in healthcare organisation, vaccination coverage and social context.^1,2^ Swedish data are therefore needed to improve counselling, follow-up and tailored interventions.

The aim of this study was to assess HRQoL in Swedish patients with genital herpes or genital warts, compare their scores with Swedish population norms, and evaluate demographic and clinical factors associated with quality of life.

## Material and methods

This cross-sectional study was conducted at the STI clinic in Region Västerbotten (Umeå, Sweden). Data were collected during routine clinical visits between November 2023 and September 2025. Inclusion criteria were adults (≥18 years) with a clinician-confirmed diagnosis of genital herpes or genital warts. Patients were eligible if they could complete the questionnaire in Swedish; this was assessed by the treating clinician during the visit. The questionnaire was completed at the end of the clinical visit. The study was exploratory and included all eligible patients during the inclusion period. The study was approved by the Swedish Ethical Review Authority (Dnr 2023-05230-01). All participants provided written informed consent.

Clinical and demographic data were obtained from questionnaires, including e.g., educational level, relationship status, recurrence frequency during the past 12 months, presence of current lesions, and treatment. HRQoL was assessed using the RAND-36 questionnaire, which includes eight domains scored from 0 to 100, with higher scores indicating better health.^5^ Global QoL was measured using a visual analogue scale (QoL-VAS). Psychological distress was assessed using the Depression Anxiety Stress Scales, 21-item version (DASS-21).^6^ An additional visual analogue scale (VAS) was used to assess sexual life. All VAS scores were recorded on a 0–10 scale.

Statistical analyses were performed using IBM SPSS Statistics version 30. DASS-21 subscales and VAS ratings (global quality of life and sexual life) were analysed using Mann–Whitney U tests and reported as median (interquartile range, IQR). RAND-36 domain scores were analysed as mean (standard deviation, SD) to allow alignment with age-matched Swedish population norms.^7^ Comparisons between diagnostic groups were performed using Welch’s t-tests, and comparisons with age-matched Swedish norms were conducted using one-sample t-tests. No correction for multiple testing was applied.

## Results

A total of 91 patients were included, 45 with genital herpes and 46 with genital warts. The genital warts group consisted predominantly of men (72%), whereas women were overrepresented in the genital herpes group (76%). Median age was 31 years in the genital herpes group and 37 years in the genital warts group, and about half of the genital herpes group and about two-thirds of the genital warts group reported having a stable partner as shown in Table 1.

**Table 1.** Characteristics and disease-related variables among participants (*n* = 91) with genital herpes (GH) and genital warts (GW).

Variable	Genital herpes (*n* = 45)	Genital warts (*n* = 46)	*p* value
Sex			
Male, *n* (%)	11 (24)	33 (72)	
Female, *n* (%)	34 (76)	13 (28)	
Age (years) median [IQR]	31 [26-45]	37 [29-47]	
Education level			
Primary school, *n* (%)	0 (0)	3 (7)	
High school, *n* (%)	22 (49)	23 (50)	
University/college, *n* (%)	23 (51)	20 (44)	
Employment status			
Employed, *n* (%)	24 (53)	35 (76)	
Student, *n* (%)	15 (33)	6 (13)	
On sick leave, *n* (%)	3 (7)	4 (9)	
Unemployed, *n* (%)	3 (7)	1 (2)	
Stable/regular sex partner			
Yes, *n* (%)	22 (49)	28 (64)	
Disease characteristics			
Current symptoms, *n* (%)	17 (45)	43 (98)	
Duration of symptoms ≤1 year, *n* (%)	15 (33)	23 (50)	
DASS-21 and QoL-VAS scores			
DASS depression, median [IQR]	6 [4–14]	6 [0–15]	0.267
DASS anxiety, median [IQR]	6 [2–8]	2 [0–8]	0.161
DASS stress, median [IQR]	12 [6–21]	9 [4–18]	0.158
Quality of life VAS (0–10), median [IQR]	7 [5–8]	7 [6–8]	0.860
Sex life VAS (0–10), median [IQR]	5 [3–8]	7 [5–8]	0.125

• Background variables are presented descriptively. Outcome variables are analysed using Mann–Whitney U tests, with *p*-values shown for these comparisons.

Almost all patients with genital warts had visible lesions at the visit (98%), whereas 45% of patients with genital herpes did so. The median number of symptom recurrences during the past year was 4 (IQR 2–7) among patients with genital herpes.

Duration of symptoms ranged from 0 to 36 years, with a median duration of 3 years for genital herpes patients and 1 year for genital warts patients. Clinical characteristics are summarised in Table 1.

Psychological and VAS outcomes are summarised in Table 1. No statistically significant differences were observed between the groups for depression, anxiety, stress, global quality of life or sexual life (all *p* > 0.12).

Differences between the diagnostic groups were observed in HRQoL assessed with the RAND-36. Patients with genital herpes reported significantly lower scores than patients with genital warts in the domains vitality and social functioning. Comparisons of HRQoL measured with RAND-36, among patients with genital herpes and genital warts (Table 2).

**Table 2.** Health-related quality of life outcomes in patients with genital herpes (GH) and genital warts (GW), and comparison with population norms based on Swedish RAND-36 norms.^7^

Outcome	Genital hepres	Genital warts	p-value: (Genital herpes vs Genital warts)	Genital herpes vs norm	Genital warts vs norm
RAND-36 domains (0–100)					
PF – physical functioning	90 ± 17	92 ± 12	0.556	n.s.	n.s.
RP – role physical	73 ± 37	83 ± 31	0.171	n.s.	n.s.
BP – bodily pain	72 ± 25	80 ± 21	0.110	n.s	n.s.
GH – general health	59 ± 23	62 ± 22	0.525	↓	n.s
VT – vitality	46 ± 20	56 ± 22	0.034	↓	n.s.
SF – social functioning	68 ± 27	79 ± 23	0.048	↓	n.s.
RE – role emotional	50 ± 41	59 ± 43	0.307	↓	↓
MH – mental health	64 ± 18	71 ± 19	0.064	↓	n.s.

• Values are presented as mean ± SD to align with published RAND-36 norms.

• Arrows indicate statistically significant deviation of the study population from population norms, *p* < 0.05.

• n.s = not significant (*p* > 0.05).

Compared with Swedish population norms, patients with genital herpes showed substantial impairments in multiple RAND-36 domains, with differences of 15–27 points for vitality, social functioning, role emotional and mental health (all *p* < 0.001). A lower score was also observed for general health (9 points, *p* = 0.015). Genital warts patients showed more limited deviations from norms, with a lower score in and role emotional (18 points, *p* = 0.008), while other domains were close to reference levels (Table 2).

## Discussion

This cross-sectional study shows that Swedish patients with genital herpes and genital warts report poorer HRQoL than population norms, while DASS-21 scores indicated elevated psychological symptoms, particularly among patients with genital herpes. Mental and social dimensions of the RAND-36 were most affected in patients with genital herpes, whereas deviations in the genital warts group were smaller and mainly related to emotional functioning.

The groups differed in sex distribution, which limits conclusions about whether observed HRQoL differences reflect diagnosis or underlying sex-related factors. However, differences in disease characteristics may help to explain the overall pattern. Genital herpes is a lifelong infection characterised by recurrences and uncertainty regarding asymptomatic transmission, which may generate persistent worry, guilt and relational concerns.^1^ Such long-term emotional strain may affect mental health and sexual well-being even in periods without visible symptoms. In contrast, genital warts, although stigmatised and sometimes recurrent, are often perceived as more treatable and temporally limited. Despite presenting with more visible lesions, genital warts patients reported better mental HRQoL than GH patients, suggesting that chronicity and unpredictability may be more burdensome than the presence of lesions at a single healthcare visit. The results are consistent with international literature showing that both genital herpes and genital warts can negatively influence quality of life, particularly through stigma, recurrent symptoms and fear of transmission.^1,2,3,4^ By integrating Swedish normative data for RAND-36^7^, the study offers context-specific insights relevant to clinical practice in Sweden.

The study could not find any significant differences in psychological distress, assessed with DASS-21, between the patient groups. There are no Swedish population norms for DASS-21 currently available. Compared to a British study presenting norms on DASS-21 in a general adult population,^8^ patients with genital herpes in our study had higher scores for depression, anxiety and stress, whereas the genital warts group showed smaller deviations from presented norms.

The study has limitations, including its cross-sectional design, single-centre recruitment and small sample size, which reduce generalisability. Recruitment from a sexual health clinic may further limit generalisability, as this population may differ from the broader patient population in terms of health-seeking behaviour, and selection bias cannot be excluded. In addition, self-report measures are subject to reporting and social desirability bias, and we did not collect data on prior mental health history or HPV vaccination status. Because of the modest sample size and substantial sex imbalance, adjusted multivariable analyses were not feasible, and residual confounding remains a possibility.

Strengths include the use of validated instruments, clinically confirmed diagnoses with data collected in routine care, reflecting real-world practice.

Clinically, the findings underscore that both genital herpes and genital warts require attention to psychological and relational aspects of care. Genital herpes patients may particularly benefit from screening for depression and anxiety, clear information about recurrence patterns, and discussion of suppressive antiviral therapy when recurrences cause psychological burden, as highlighted in a recent study.^9^ For genital warts, symptoms should not be trivialised, as reduced general health and emotional functioning may persist despite lesion-directed treatment. Counselling and patient education remain essential, and the anticipated long-term impact of HPV vaccination on future genital warts burden should be considered in follow-up planning and resource allocation. In summary, both conditions are associated with reduced HRQoL compared to population norms, with the most pronounced impact in the genital herpes group. Both genital herpes and genital warts should be viewed as potential long-term stressors affecting mental well-being, relationships and sexual health.

## Data Availability

The datasets generated and analysed during the current study are available from the corresponding author upon reasonable request.*
